# Biomedical Data Repository Concepts and Management Principles

**DOI:** 10.1038/s41597-024-03449-z

**Published:** 2024-06-13

**Authors:** Dawei Lin, Matthew McAuliffe, Kim D. Pruitt, Anupama Gururaj, Christine Melchior, Charles Schmitt, Susan N. Wright

**Affiliations:** 1 National Institute of Allergy and Infectious Diseases (NIAID), National Institutes of Health, Bethesda, Maryland, USA; 2 Center of Information Technology (CIT), National Institutes of Health, Bethesda, Maryland, USA; 3 National Center for Biotechnology Information, National Library of Medicine, National Institutes of Health, Bethesda, Maryland, USA; 4 Center for Scientific Review (CSR), National Institutes of Health, Bethesda, Maryland, USA; 5 National Institute of Environmental Health Sciences (NIEHS), National Institutes of Health, Durham, North Carolina, USA; 6 National Institute on Drug Abuse (NIDA), National Institutes of Health, Bethesda, Maryland, USA

**Keywords:** Genetic databases, Genetic databases

## Abstract

The demand for open data and open science is on the rise, fueled by expectations from the scientific community, calls to increase transparency and reproducibility in research findings, and developments such as the Final Data Management and Sharing Policy from the U.S. National Institutes of Health and a memorandum on increasing public access to federally funded research, issued by the U.S. Office of Science and Technology Policy. This paper explores the pivotal role of data repositories in biomedical research and open science, emphasizing their importance in managing, preserving, and sharing research data. Our objective is to familiarize readers with the functions of data repositories, set expectations for their services, and provide an overview of methods to evaluate their capabilities. The paper serves to introduce fundamental concepts and community-based guiding principles and aims to equip researchers, repository operators, funders, and policymakers with the knowledge to select appropriate repositories for their data management and sharing needs and foster a foundation for the open sharing and preservation of research data.

## Introduction

Public demand and policy support for open data and open science are growing. In August 2022, the U.S. Office of Science and Technology Policy (OSTP) issued a memorandum^1^ directing federal agencies to develop plans for the immediate release of federally funded research results, including publications and data, without an embargo period. This builds upon a 2013 memorandum that had directed federal agencies to develop similar plans but suggested a 12-month post-publication embargo period as a guideline^2^.

Similarly, the final NIH Data Management and Sharing Policy (NIH DMS Policy)^3^, which went into effect in January 2023, requires the development of data management and sharing plans for all NIH-supported research and expects researchers to maximize appropriate sharing of scientific data. Enabling such broad culture change and policy implementation requires reliable, secure, and trustworthy data repositories.

Data repositories are essential for managing, preserving, and sharing research data, and have become indispensable resources for biomedical research communities. They provide a central location for researchers to deposit their data, and they offer a variety of services to make it easy for others to find, access, and use the data. Data repositories also play a vital role in promoting data sharing and collaboration, and they help to ensure that research data is preserved for future generations.

The objective of this paper is to explain the functions of data repositories, set appropriate expectations for their services, and introduce methods to evaluate their capabilities in meeting the evolving needs of users. Our approach to achieve this objective involved in participating in discussion groups, hosting workshops, conducting interviews, performing a Metrics and Lifecycle survey, analyzing the landscape analysis of biological data repository lifecycle management, and synthesizing information from that work as well as from the literature, websites, and from community groups including the Research Data Alliance (RDA)^4^. Our intention is for these results and collated information to advance the work of diverse users including but not limited to researchers who are choosing a data repository, data repository managers, publishers, policy experts, and funders.

## Results

We define fundamental concepts and introduce categories and access types of data repositories and their operations within the context of biomedical research. Additionally, we present community-based guiding principles and best practices for utilizing and assessing repository management.

### Concept introductions

This section provides an overview of the concepts and terminology used to describe data repositories and different types of data access approaches. The definitions provided here are not exhaustive but represent the major categories of data repositories and data access approaches in the biomedical data ecosystem. Terms and definitions presented in this article were formed with extensive consideration of publicly available information including community perspectives gleaned from publications and internet documents, workshop engagements, and author deliberations (see Methods).

#### Data resources

This paper focuses on the “data repository” as it aligns with the language utilized in the NIH DMS Policy^3^, the NIH Data Science Strategic Plan^5^, the NIH Desirable Characteristics of Data Repositories^6^. While we briefly introduce the concept of knowledgebase, which is a closely related data resource, our discussion centers on data repositories. Nonetheless, the principles and approaches discussed in this paper can generally be applied to knowledgebases.

- **Dataset**: A collection of discrete, related data items that may be accessed individually or in combination or managed as one digital entity. Both data repositories and knowledgebases may hold datasets and provide access to the dataset and/or individual data items. For example, an individual nucleotide sequence is a data item, and a collection of sequences generated from a research study comprise a dataset. A dataset may also be a collection of multiple types of data that are related scientifically by a study or project.
- **Data resource:** A data resource refers to any collection of data that is systematically organized and managed to serve a specific purpose. Its primary function is to support a wide array of activities, such as research, decision-making, and planning. Data resource is a general term that encompasses data repositories, knowledgebases, datasets, or public websites that provide access to and views of data. In the context of the NIH’s vision for a modernized biomedical data ecosystem, as outlined in the NIH Strategic Plan for Data Science^5^, data resources are further categorized by NIH into two distinct types: data repositories and knowledgebases^7^.
- Biomedical **data repository:** Systems that accept submissions of relevant data from the research community to store, organize, validate, archive, preserve, and distribute the data, in compliance with principles and regulations. Data repositories may host data for a specific domain of science or may host data from multiple domains. Data repositories hold data that researchers make available for others to reuse. Data repositories may be open to the public or restrict access to protect privacy and confidentiality of data from human research participants. Examples are the Protein Data Bank^8^, GenBank®^9^, and ImmPort^10^.
- Biomedical **knowledgebase**: Systems that extract, accumulate, organize, annotate, and link a growing body of related information that is related to, and relies on, core datasets managed by data repositories. Unlike data repositories, knowledgebases may not accept direct submissions of research data but instead focus on extracting meaningful knowledge from existing information sources. While most knowledgebases are open access, some community knowledgebases (e.g., OMIM^11^, TAIR^12^) have established a donation or subscription approach to maintain services. Knowledgebases may have different focuses, such as a disease, an organism, a gene type, or other categories. Examples are UniProt^13^, ClinVar^14^, and Reactome^15^.

#### Types of data repositories

Data repositories are commonly categorized into four types: domain-specific, project-specific, institution-specific, and generalist. While there may be overlapping services provided by these repositories, each type serves a distinct purpose. For example, data held in an institutional repository could be specific to a project or could be specific to a domain of data while data held in a project-specific repository could be of a specific domain or could alternatively fit into a generalist repository.

- **Domain-specific repositories:** These repositories store data of a specific type (e.g., protein structure, nucleotide sequence, clinical data) or discipline (e.g., cancer, neurology). They often form a nexus of resources for their research communities interested in these specialized data.
- **Generalist repositories**: These repositories store data of multiple types and disciplines, accepting data regardless of its type, format, content, disciplinary focus, or research institution affiliation. NIH has established agreements with several generalist repositories under the NIH Generalist Repository Ecosystem Initiative (GREI)^16^.
- **Project-specific data repositories:** These repositories store domain-specific data generated from a project or collaboration (e.g., NIH All of Us^17^) and enable data sharing and reuse by making the project-specific data available for reuse by other projects or researchers. This is not to be confused with a project data coordinating center (DCC), which facilitates the project collaboration, curation, and data analysis but does not serve as a repository as data are not widely available for reuse by other researchers. Note that a DCC may also later facilitate submission of the project data to a data repository.
- **Institutional repositories:** These repositories store data primarily created by members of an institution or a group of institutions, such as principal investigators (PIs), postdocs, and students. This category addresses the needs of the institution’s staff and may serve to collect data from one-to-many projects, and, depending on the institutional mission, may function as a domain-specific or generalist repository.

The various types of repositories have emerged in response to the diverse needs of researchers to share data, which includes complying with policy requirements, supporting ongoing collaborations, and promoting open science and FAIR (Findable, Accessible, Interoperable, and Reusable) Principles^18^. The NIH DMS Policy^3^ emphasizes the importance of good data management practices and encourages data management and data sharing that reflect practices within research communities. Data management and sharing should reflect practices consistent with FAIR Principles to be most beneficial. NIH-supported and NIH-managed repositories are the building blocks of the NIH data ecosystem and one of the primary mechanisms by which NIH makes the results of federally funded data available to the research community and the public. Federally funded data repositories should adopt the OSTP Desirable Characteristics of Data Repositories^19^ and should align with community standards such as the TRUST (Transparency, Responsibility, User focus, Sustainability, and Technology) Principles^20^ and CARE (Collective Benefit, Authority to Control, Responsibility, and Ethics) Principles^21^.

#### Common characteristics of data repositories

**Fig. 1:**
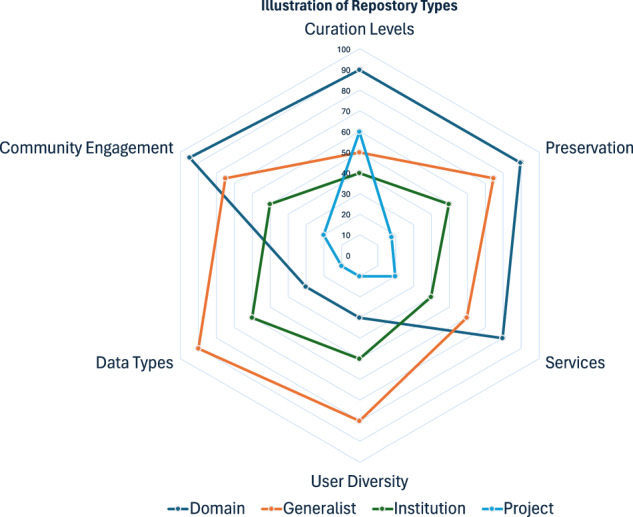
Illustration of Repository Types. This figure is an illustration of different repository types, which often prioritize different aspects of data management and sharing. Each ring with a distinct color represents a repository type, namely, blue for Domain-specific repository, orange for Generalist repository, green for Institutional repository, and cyan for Project-specific repository. The diagram provides a generic impression of prioritization and should not be viewed as universally representative of specific repositories. The priority and emphasis are assigned on a 0–100 scale. 100 has the most priority. The digital scale is for illustration only. For example, domain-specific repositories tend to have in-depth curation and close interaction with their user community, while generalist repositories can accommodate many data types and diverse users.

Figure 1 illustrates elements of data repositories based on six major characteristics and maps the different types of data repositories onto these features: community engagement, curation, preservation, user diversity, services, and data types.

- **Community Engagement:** The extent of a repository’s involvement with its community. Project- and domain-specific repositories, for instance, may rely on external advisory boards composed of subject matter experts to ensure the content of data aligns with broad perspectives of relevant research fields. Project-specific repositories may also have extensive communications with project-specific stakeholders, within the scope defined for the project. They also employ transparent community engagement processes to inform key stakeholders of adopted and upcoming format changes, minimizing disruption to the domain data ecosystem, including tools that use or produce data in those formats. The project repositories serve and consult a focused community associated with the projects of interest. In contrast, generalist or institutional repositories may demand less user engagement at the level of content, as they serve a more diverse and larger community where users may have varying purposes and approaches to using data.
- **Curation:** The process of employing various standards and best practices to transform data into meaningful organized, structured, and computable forms^22,23^. Data curation involves quality assurance (QA) and quality control (QC) for data accuracy, along with cleaning, integration, and annotation for clarity. Additionally, it includes normalization for consistency, classification for organization, management of data licensing to adhere to legal standards. Generalist and institutional repositories may use metadata standardization to boost findability and accessibility, while domain and project repositories may apply extra efforts to ensure data adherence to field-specific standards for increased interoperability and reusability.
- **Preservation:** The extent to which the data repositories invest resources in archiving data for long-term use, including adapting to evolving user needs, changes in storage technology, and changes in media formats. Preservation is a mandatory responsibility that is shared by all repositories. The project or institutional repository may have limited lifespan due to user base and mission.
- **User Diversity:** Generalist repositories are designed to accommodate a diverse audience, offering resources and support for users across multiple disciplines and skill levels. They aim to be inclusive and accessible, providing a foundation for both introductory learning and advanced research. Conversely, domain and project repositories offer a platform for the in-depth exchange of knowledge and resources tailored to specific fields, projects, or technologies. They serve as specialized hubs for experts and practitioners seeking detailed information and collaboration within their areas of expertise.
- **Services:** Services that support the objectives and obligations of a repository. All repositories provide core services such as ingestion (intake) of data, data management, preservation, archival storage, administration, access. Moreover, domain-specific ones may provide additional services, such as analysis and visualization tools, external links to or searchability with other data resources, and educational materials to meet their specific needs. Subsequently, journals often collaborate with open access generalist repositories to align manuscript and data submission processes, thereby ensuring that the underlying data is both accessible and discoverable^24,25^.
- **Data types**: Categories of digital assets that are managed by a repository. For example, domain-specific repositories are designed to collect data related only to a particular modality, technology, or research domain. In contrast, generalist and institutional repositories are not subject-focused and may archive data and other digital objects with no other appropriate place to go, such as data from rapidly advancing technologies that are still too new to have a domain-specific repository.

#### Data access types

Data access types refer to the different ways in which users can access data from a repository. The type of data access that is most appropriate for a particular repository will depend on a number of factors, including the nature of the data, the intended audience, and the funding model. There are five main types of data access:

- **Open access**: Data is freely disseminated with no requirements for user to register, log in, pay, or provide justification to access the data. Data in open access repositories is often considered to be easily findable, accessible, and equitable, as access is open to all interested parties, including those working in public health, research, education, and the general public.
- **Registration required**: Data can be accessed by anyone who first registers and logs into the resource. Registration can benefit data repositories as they can have richer information about their user base and patterns of data access.
- **Controlled access**: Access to the data, typically originating from human studies, is controlled through measures such as requiring data requesters to verify their identity and the appropriateness of their proposed research use. Typically, a committee reviews the application to ensure that eligibility requirements are met before granting permission to access the data. Data enclave is a subcategory of controlled access provides additional secure access controls as it defines a system boundary through which data cannot be downloaded (e.g. All of Us^17^ or N3C^26^)
- **Pay to access or donation suggested**: Data submission or access models that request funds to support the sustainability of the resource. In some cases, users must pay to register for data submission or data access whereas in other cases the repository has partial funding and requests donations from the community to fully meet their costs. It is worth noting that charging fees for data access conflicts with the OSTP descriptions of desirable characteristics of data repositories^19^ and principles of equitable data access. The NIH and OSTP encourage free and easy data access^6,19^, although paid funding models may be justified given the context that organizational and technical sustainability are also desired characteristics.
- **Closed access**: Access typically is not provided to general users. Closed-access proprietary repositories may be developed by commercial interests, for example to support the research needs of pharmaceutical or companies.

### Principles & properties

Recognizing the value of data involves tackling complex technical and social challenges. Various stakeholders, including those from academia, industry, funding agencies, and scholarly publishing, introduced the ***FAIR Data Principles***^18^ to support effective data management and stewardship and improve machine actionability. Utilizing a similar community-driven approach, the ***TRUST Principles***^20^ unified data preservation and repository communities, underlining best practices in repository operations and their sustainability. Similarly, the ***CARE Principles for Indigenous Data Governance***^21^ developed by the Global Indigenous Data Alliance (GIDA)^27^ highlights the importance of equitable data usage and addresses the rights of indigenous people, tribal sovereignty, and the control of their data, helping in part to inform the complex issue of releasing sensitive data. The NIH has developed supplemental information for assisting researchers in developing appropriate Data Management and Sharing Plans when working with American Indian/Alaska Native Tribes that may also be relevant for selecting or establishing repositories^28^. Collectively, the three principles cited above complement one another and address technical, operational, and social challenges, respectively.

In addition to the community-developed principles cited above, the U.S. Government developed guidance for data sharing by federal agencies. The NIH and OSTP recommend that researchers select repositories that meet the Desirable Characteristics for Repositories^6,19^. Repositories should collect and monitor metrics that summarize how well they are doing in meeting these principles and serving their user communities. An NIH report provides information on metrics to collect and the state of metric usage among repositories^29^. Repository directors and stakeholders also should be cognizant that repositories are not static entities, but rather resources that require lifecycle management in response to shifting needs of the communities being served. Section 3.4 will further discuss the lifecycle of repositories.

**Fig. 2:**
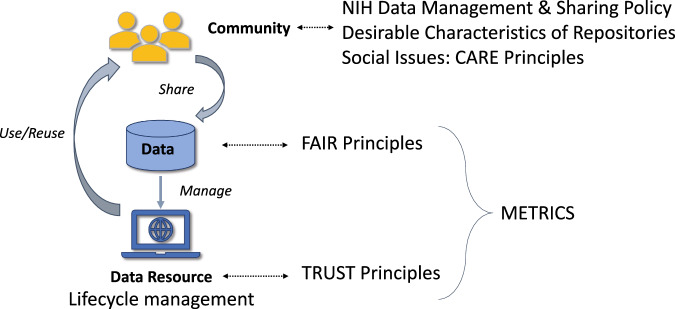
Data Ecosystem Principles and Practices. Community awareness of the NIH Final Data Management and Sharing Policy and the Desirable Characteristics for Repositories, and social considerations for community concerns, such as CARE Principles, are important to aid in assessing and selecting repositories. FAIR and TRUST Principles inform data and repository practices respectively. FAIR speaks to data and metadata properties and TRUST addresses specifically repository operation. They both can be assessed with metrics throughout the lifecycle of a repository.

These notable principles and their relationship to data repositories are depicted in Fig. 2.

#### Desirable characteristics for repositories

With a focus on promoting transparency, reproducibility and input from the community, NIH has identified a set of desirable characteristics that data repositories should embody^6^. These characteristics serve as guidelines for establishing robust repositories that ensure the access, management and integrity of data and enhance its discoverability, interoperability, and reusability.

The NIH Desirable Characteristics of a data repository^6^ include the use of:

- **Unique Persistent Identifiers** for datasets to support discovery, reporting, and assessment.
- **Metadata** to describe datasets to enable discovery, reuse, and citation.
- **Common Format** for datasets and metadata, preferably non-proprietary, community based.
- **Provenance** to record the origin, chain of custody, and modifications to data/metadata.

Repositories should ensure:

- **Curation and Quality Assurance** to enhance the accuracy and integrity of data/metadata.
- **Free and Easy Access** to data/metadata consistent with limits needed for sensitive data such as tribal sovereignty and controlled-access human data.
- **Broadest and Measured Reuse** of data/metadata with attribution and citation.
- **Security and Integrity** of data/metadata using documented and appropriate measures.
- **Confidentiality** of sensitive data using documented and appropriate measures.

Repositories should provide:

- **Clear Usage Guidance** on terms for access and use of data.
- **Retention Policy** that documents policies for retention of data.
- **Long-term Sustainability** through policies and procedures that maintain integrity, authenticity, and availability of metadata and data.

For repositories holding human data, additional desired characteristics include:

- **Fidelity to Consent** using documented procedures to ensure access and use of data are consistent with any restrictions imposed by participant consent.
- **Restricted Use Compliant** enforcing restrictions and ensuring access and use of data are consistent with participant consents.
- **Privacy** protecting human subjects’ data from inappropriate access.
- **Plan for Breach** handling security breaches and unauthorized access to data.
- **Download Controls** controlling over data downloads including audits of access.
- **Violations** addressing the violations of terms of use by user or mismanagement by the repository.
- **Request Review** establishing and maintaining a transparent process.

#### FAIR Principles

The FAIR Data Principles^18^ aim to increase the value of data by enabling researchers to readily find data and to reuse the data. Such efforts are helpful for secondary analysis, conducting replication studies, performing meta-analysis across study data, and generating new integrated datasets, etc. FAIR calls for research data shared through a repository to be:

- **Findable:** data/metadata are assigned with global, persistent unique identifiers, data is described with linked metadata and data/metadata are registered or indexed in a searchable resource.
- **Accessible**: data/metadata are retrievable with requirements for authentication/authorization as needed. Metadata should be accessible even when data are no longer available.
- **Interoperable**: data/metadata use a formal and broadly applicable language and ontology for knowledge representation with qualified references to other metadata/data.
- **Reusable:** data/metadata are richly described with accurate, relevant attributes, released with a clear and accessible data usage license, have detailed provenance, and meet domain-relevant community standards.

#### TRUST Principles

The TRUST Principles^20^ focus on activities and best practices for management of data repositories. The objective is to ensure the long-term accessibility of the data to its users while maintaining desirable properties, being as open as possible or as closed as necessary and adhering to the principles of FAIR data if applicable.

TRUST calls upon repositories to demonstrate the following practices:

- **Transparency:** ensure their mission, scope, terms of use, preservation timeframe, and other pertinent features and services are transparently declared.
- **Responsibility**: act as responsible stewards of their data and the community they serve, including by adhering to community standards and expectations, providing needed services, and providing appropriate safeguards on data.
- **User Focus**: support the needs of their target user community and demonstrate adherence through actions such as providing metrics and monitoring and responding to the community.
- **Sustainability:** ensure uninterrupted access to data for current and future users, which may include planning risk mitigation, business continuity, securing funding, and providing for long-term preservation.
- **Technology:** ensure the repository is providing secure, persistent, and reliable services and using relevant and appropriate technologies and practices and addressing security threats.

The TRUST Principles in their non-technical formulation facilitate communication and thus impact stakeholders both within and outside the data user community. When data repositories, repository funders, and data creators adopt FAIR Principles and implement the TRUST Principles, repository users benefit directly through continuingly improved capabilities for efficient and effective reuse of data.

#### CARE Principles

The CARE Principles^21^, developed by the GIDA^27^, address concerns over secondary use of data related to Indigenous communities who want to protect their rights and interests, and provide benefit to the communities providing the data, while allowing opportunities for research. The CARE Principles address concerns that include:

- **Collective Benefit:** ensure that repositories and data function to support the populations they represent and that the use of data reflects community values.
- **Authority to Control:** recognize the rights and interests of a community to govern community data, including determination of data governance policies and protocols, involvement in stewardship and access decisions, and rights to access and use community data to support the governance and self-determination of the community.
- **Responsibility:** recognize the need to foster respectful relationships with the communities that data derive from, including investing in capabilities and capacity for the community and generating data that is grounded in the languages, worldviews, and lived experiences of Indigenous Peoples.
- **Ethics:** recognize that repositories and data holders should minimize harm and maximize benefit from use of community data, promote justice, and allow for future data use. This includes representation and participation from the community to ensure use reflects community values and ethics.

### Repository management best practices

#### Repository operations

Repository operations are the processes and activities that are necessary for a data repository to function. These operations include accepting and processing data submissions, securely storing and preserving the data, and providing interfaces for accessing the data.

The Open Archival Information System (OAIS) reference model is an International Organization for Standardization (ISO) standard (ISO 14721)^30^ that is widely used to describe repository operations in a manner that is not limited to a specific domain or discipline^31^. The application of the OAIS model for preservation of biomedical data has been discussed^31^ and informs the design and operations of many repositories. Adhering to such a standard helps repository professionals develop community consensus on guiding principles, best practices, and recommendations.

The OAIS model outlines a repository system consisting of six components: data ingest, archival storage, data management, administration, preservation planning, and data access. The OAIS model also serves as the foundation for repository certification standards, data preservation tools, and software products. As a community standard, it undergoes updates every five years to accommodate the ever-changing needs of the digital repository and archive landscape.

A well-structured and managed repository operation helps ensure reliable data preservation and provision of useful services to end users who rely on the repository for data sharing and data access to support research.

Users of a data repository should understand the operational aspects of a repository, such as the types and scope of data that the repository accepts, the necessary metadata and format for data curation, the data intake process, data validation and curation procedures, and the timeline to generate a publicly shareable dataset. Additionally, repositories require time to index data for searches and coordinate with their partners. Consequently, data are often released in batches and at specific intervals. It would be prudent for users to familiarize themselves with such schedules. Coordinating these with the timing of publication may be necessary, given that specific journal policies, funding agencies or sponsorship rules may mandate data sharing upon publication or the end of an award period.

#### Repository trustworthiness and certification standards

The trustworthiness of a repository’s operation is based in part on the process to provide quality data, reliable access to data, and sustainable practices. Together these enhance scientific reproducibility by ensuring the data are collected, organized, and stored using agreed-upon, established criteria.

Over the years, community-based standards have emerged to assess the quality of repository operations. Some repositories opt for certification through independent verification and assessment of their publicly accessible evidence and documentation, while others choose to conduct self-assessment. Some repositories endorse the TRUST Principles as a means of demonstrating their dedication to offering trustworthy services.

Table 1 lists three major standards to certify repositories: CoreTrustSeal, nestor Seal, and ISO 16363.

**Table 1:** This table summarizes three sources of certifications for repositories.

Organization	CoreTrustSeal^36^	nestor Seal^34^	ISO: International Organization for Standardization^32^
Number of Requirements	16	34	100+
Standards	Requirements 2023–2025^78^	DIN 31664^79^	ISO 14721 (OAIS)^30^
			ISO 16363^80^
			ISO 16919^81^
			ISO 17021^82^
Audit Process	Self-assessment + independent peer review	Self-assessment + independent peer review	ISO certified audit with accredited auditors
Certification Cost*	€3000	€500	$20,000
Designation	CoreTrustSeal certification	nestor Seal for Trustworthy Digital Archives	ISO certification
Certification lifespan	3 years	Indefinite	3 years
Number of Certified Repositories (as of May 2024)	160+	4	1

*Approximate estimated cost as of May 2024, subject to change by certifying organization

The major assessment areas in common for all three certification sources include:

- Organization
- Management of intellectual entities and representations
- Technology infrastructure and security

The differences reside in how the certification audit is performed and the number of factors and details that are evaluated in the audit.

Ultimately, the choice of certification depends on whether and how much a repository is willing to invest in obtaining independent certification. Individual repositories may choose to pursue certification from one of the available providers, considering various factors such as cost, requirements, funder perception, and the acceptance or recognition of the certifying entity. One benefit of external certification is assurance to users of the repository’s good operational practices. Certification costs include both the certification fees themselves, time, and personnel efforts spent on preparing materials for the certification audit. The latter can be substantial because certifying a repository can take months, depending on the repository’s maturity, level of existing documentation, and overall readiness to provide the materials needed for the audit. Depending on the organization used for certification, repositories may need to undergo re-assessment periodically. Changes in technology and user needs can also drive re-certification.

So far, the ISO^32^ has certified one repository, the U.S. Government Publication Office (GPO) govinfo repository^33^; nestor Seal^34^ has certified four German repositories^35^; and CoreTrustSeal^36^ has certified more than 160 repositories^37^ across several scientific disciplines and across the globe.

#### Repository metrics

The continued operation and success of a repository relies not only on the quality and accessibility of the data stored within it, but also on the broader scientific impact of the use of the data. Repository managers have an interest in demonstrating and quantifying the impact of their repositories for past, present, and future research endeavors. This impact could be quantified based on different perspectives or characteristics of the repository and the data it contains. Metrics provide systematic parameters for evaluating the cost and benefits (return on investment) of a repository to the various stakeholders including managers, research institutions, funding agencies, and research communities. Additionally, there are efforts to standardize the requirements of Biodata Resources via Global BioData Coalition^38^.

While data metrics are an important aspect of repository metrics, the two can be differentiated. Repository metrics are aggregate measures of access and impact of the provided services encompassing all the data held and indicate the use, value, and impact of the repository as a whole. In contrast, data metrics are a granular measure of individual data (or a defined dataset) reuse, value, and impact. Data metrics provide insights into the value of datasets for reuse or alignment to FAIR Principles over time. Make Data Count is another global, community-driven effort aimed at developing standardized metrics to evaluate and acknowledge the impact of research data^39^.

As noted in the methods section and described in detail in the report^29^ [12] produced by the Metrics for Data Repositories and Knowledgebases Working Group, biomedical repositories were reviewed via multiple techniques (including discussions with repository managers and a survey to repository stakeholders). Based on this assessment, a list of metrics that are most commonly collected and used by repository managers were identified^29^, as shown in Table 2. The metrics are grouped into several broad categories including (from most to least commonly collected):

- User Behavior Characteristics
- Scientific Contribution/Impact
- Repository Operations

**Table 2:** List of commonly collected repository metrics.

Categories	Metrics	Description
User Behavior Characteristics	Number of users	Number of users who use the services for the data (visualization, e.g.)
	Page views	Clicks, page scrolling, mouse movement/pointing
	Downloads	Number of downloads or users downloading data, Web or FTP
	Geography	User IP address based - resolved to country/state
	New vs. Returning users	For a defined period, usually three months
	Dataset submitters	Number of data submitters
	Visit frequency	Daily, monthly, etc.
	Data access requests	How many data requests are made in a specified time period
Scientific Contribution/ Impact	Number of projects/studies	Number of projects or studies
	Number of cases/subjects	Total number of cases or subjects (e.g. individual human participant-level data)
	Total publications	Total number of publications over all the years
Repository Operations	Storage costs	Total storage cost for repository
	Cost/dataset (storage)	Cost per dataset (i.e. storage)
	Hardware costs	Total hardware costs
	Total download costs	Total download costs

The report^29^ provides a better understanding of the metrics currently used within the biomedical repository community, which can inform future efforts to objectively assess the value and impact of biomedical data repositories and, with further development, understand patterns of use across NIH-supported datasets and repositories.

#### Repository lifecycle

**Fig. 3:**
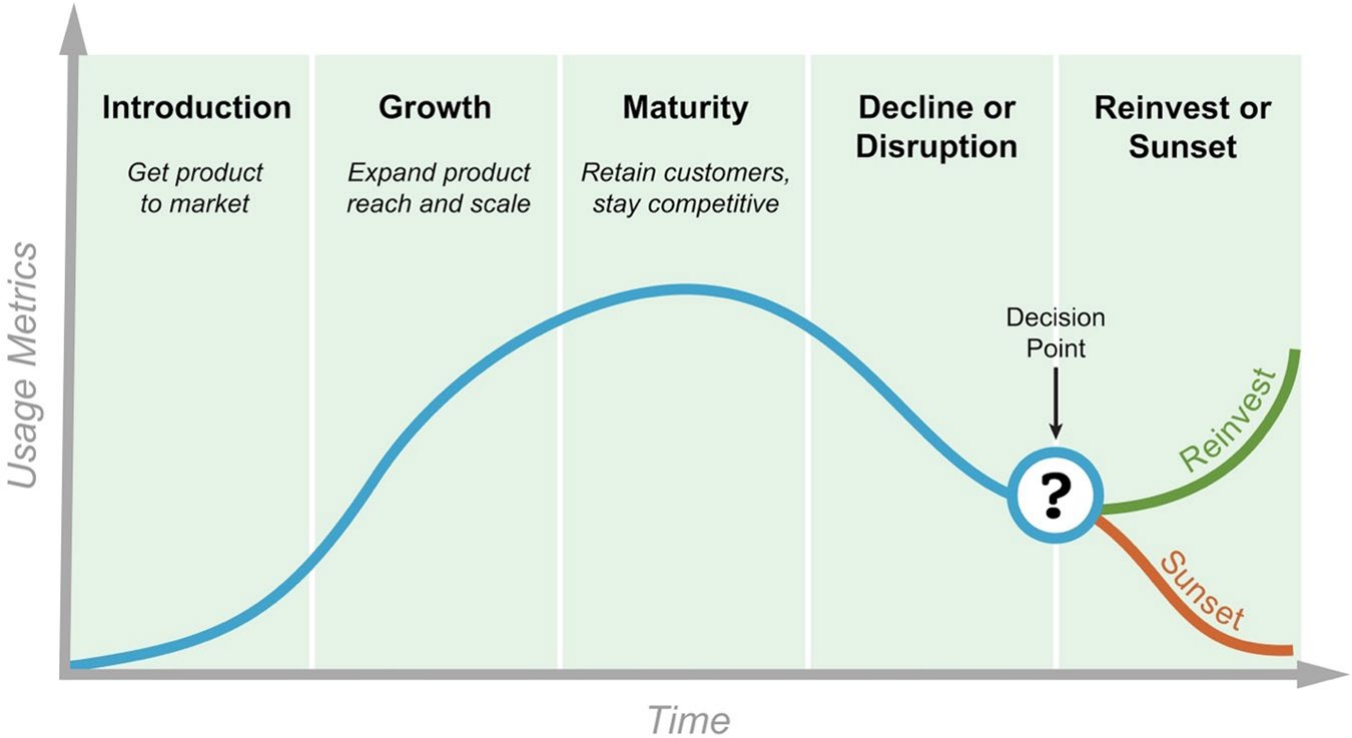
A Biomedical Repository Lifecycle Model (Image Courtesy of Bart Trawick, NLM, NIH).

To ensure long-term success and impact of data repositories, it is essential to understand their lifecycles. The NIH has developed a model that outlines the various phases of the lifecycle for both early-stage and established repositories (see Methods). This document aims to provide insights into the lifecycle of biological data repositories, highlighting key considerations and milestones at each stage (shown in Fig. 3).

1. **Introduction**: The repository lifecycle begins with the Introduction phase, where a new repository emerges or an existing resource transitions into a more structured and standardized framework. This phase involves the initial development and establishment of the repository, often in response to an unmet community need, with an emphasis on addressing governance, operational efficiency, and quality control. The initial introduction may be a minimum viable product with a partial set of features (an early-stage repository) and the goal of gathering stakeholder and community feedback on value and usability. In this phase the repository may undergo frequent feature changes in response to feedback, and overall usage rates may be low. The repository may pilot data consolidation efforts or support investigator-initiated research initiatives to lay the foundation for future growth.
2. **Growth**: Once established, repositories enter the Growth phase, where they experience an increase in usage, adoption, and community engagement. During this phase, repositories focus on expanding their data holdings, enhancing data accessibility, and improving the user experience. The repository’s value proposition and services are refined to align with the evolving needs of the research community. Repository managers continue to gather community feedback to further enhance the value of the service provided and the repository is still undergoing active development to add features. Metrics such as data size and user engagement become important indicators of success.
3. **Maturity**: As repositories reach the Maturity phase, they have achieved significant adoption and become critical research resources within the scientific community. During this phase, repositories optimize their operations, strengthen collaborations, and maintain high data quality standards. User feedback, citation and usage metrics, and the repository’s impact on research output play a crucial role in assessing its effectiveness and relevance. Continuous improvement and innovation remain essential to sustain the repository’s value over time.
4. **Decline or Disruption**: Repositories may eventually encounter a phase of Decline or Disruption. This phase can result from various factors, such as emerging technologies, changes in research practices, evolving data sharing paradigms, changing priorities of the managing organization, loss of funding or unmanageable cost increases, or acute IT and security issues. A repository may enter this phase based on declining value of the data held to user communities or other factors. It is crucial to monitor user engagement, assess the repository’s impact, and identify potential disruptions early on. Proactive measures such as updating infrastructure, exploring new data types, or fostering partnerships can help repositories reverse the decline and recover the user base if addressed early. If the decline continues the repository manager carries out a detailed assessment to understand continued relevance, availability of a similar repository, opportunities for improvement, budget, and metrics.
5. **Reinvest or Sunset**: At the Decision Point, repository managers assess the repository’s future trajectory. Based on an evaluation of the repository’s impact, usage, and sustainability, a decision is made to either Reinvest or Sunset the repository. Reinvestment involves strategic planning, incorporating feedback from the research community, redefining the repository’s vision and services to ensure continued value, and investing in new technical development to modernize the repository to meet user needs. In contrast, Sunset options include phasing out operations, transitioning data to other repositories, or archiving the repository to preserve its legacy.

Understanding the lifecycle of biological data repositories is vital for their successful development, operation, and impact. The NIH repository lifecycle model provides a framework for repositories at different stages, guiding their growth, adaptation, and decision-making processes. By considering the unique characteristics and challenges within each phase, repositories can effectively serve the needs of the research community and contribute to scientific progress in the long term. Continuous evaluation, engagement with stakeholders, and proactive measures are key to sustaining valuable biological data repositories throughout their lifecycle.

In addition to the biological data repository lifecycle described here, it is important to recognize the significance of certain repositories that have become an integral part of the national and international scientific and economic infrastructure, such as core repositories listed by the registries Elixir^40^ and Global Biodata Coalition (GBC)^38^. These repositories have undergone an Introduction and Growth phase, followed by a long Maturity phase with periodic reinvestments to continually meet the evolving needs of users, and are peer reviewed and selected as core repositories.

In addition, those repositories are characterized by their sustainability and their critical role within the research and education ecosystem^41–43^. They have become indispensable resources, relied upon by society for ongoing research and education activities, as well as meeting data sharing policies mandated by funders, publishers, and data submitters.

Notable examples of such repositories include the PDB^8^, which serves as the primary repository for protein 3-D structure data. The PDB has played a pivotal role in enabling research in structural biology and facilitating drug discovery efforts. Similarly, members of the International Nucleotide Sequence Database Collaboration (INSDC)^44^ manage essential sequence and metadata repositories, such as the National Library of Medicine’s GenBank^9^ and Sequence Read Archive (SRA)^45^. These repositories serve as crucial resources for genomic data from around the world.

These repositories sometimes combine their traditional role of supporting academic research domains with providing other critical public benefits. For example, public health agencies rely on repositories to collect and manage data for surveillance and epidemiology purposes. Examples include the Centers for Disease Control and Prevention (CDC)^46^ in the United States and comparable organizations in other nations, which collect and analyze public health data and deposit data in repositories (e.g, SARS-CoV-2 sequence data deposited in GenBank and SRA). Similarly, care providers and hospital networks, such as the U.S. Department of Veterans Affairs^47^, manage repositories that store data crucial for patient care and research, as exemplified by initiatives like the VA Million Veteran Program^48^.

These repositories represent the culmination of long-term efforts to establish robust and sustainable resources that support scientific progress and societal needs. Their existence and continued relevance underscore the importance of strategic reinvestments and ongoing utility to the scientific community, ensuring that repositories meet the requirements of current and future generations of researchers and stakeholders.

### Resources and communities for repositories

Several resources and organizations provide lists of repositories that are available for use by specific user communities. The information provided by each resource may incorporate aspects of repository metadata or policy expectations. Some organizations such as funding agencies, academic publishers, etc. provide a list of supported or recommended repositories to use for data sharing. Other organizations function as registries and simply collate and categorize data repositories as a service to the community. Additionally, specific organizations provide lists of recommended repositories including institutional repositories that serve the institutions’ community to satisfy their data-sharing needs. Below is a non-comprehensive sampling of such organizations.

#### Journal recommendations

As data sharing becomes increasingly important, several journals have instituted policies and mandates that require research data to be shared. Consequently, journal publishers may provide guidelines recommending use of specific repositories for defined data types and/or guidelines that help researchers decide how and where to store and share their data. Some of the publishers who provide such lists for recommended biomedical data repositories include: Scientific Data^49^, Elsevier^50^, PLOS^51^, EMBO Press^52^, Springer-Nature^53^, Wiley^54^, GigaScience^55^ and F1000Research^56^. Generally, the publishing community recommends sharing data with domain-specific repositories as the first option followed by institutional repositories and generalist repositories. Therefore, the recommendation lists are typically categorized along the same lines. Some publishers, such as Elsevier, also provide a general repository wherein authors can deposit their data at the time of manuscript publishing. Many professional societies support repositories in their domain area. Examples include the International Society for Advancement of Cytometry (ISAC)^57^. Most of these professional organizations are also involved in publication of journals and the society-supported repositories are primarily maintained in support of their publications.

#### Global efforts

As digital research data repositories are developed and made available to their respective communities, it becomes important to be able to search and identify research data repositories and the functionalities that they provide. This service is provided by various registries that index and aggregate metadata about data repositories to make the repositories themselves findable.

Example registries include:

- **Global Biodata Coalition (GBC):** The Global Biodata Coalition^58^ aims to provide a forum for funders to coordinate, for communities to collaborate, and for users to identify available core repository resources in the biological domain. The GBC lists core repositories through an application and evaluation process^38^.
- **Nucleic Acids Research:** Oxford University Press maintains a peer-reviewed, publication-based molecular biology database collection^59^. This collection is a compilation of databases reported in the annual Nucleic Acids Research databases issue. The collection provides links to the publications that describe the database as well as links to the various home pages and contact personnel of the databases.
- **re3data:** The Registry of Research Data Repositories (re3data) is a general global registry covering all academic disciplines^60^. This registry has been in existence for the past decade and operates under the umbrella of DataCite^61^ services. The registry publishes defined inclusion criteria for indexing, and the metadata about a resource follows an in-house schema: re3data.org Schema for the Description of Research Data Repositories.
- **FAIRsharing.org:** FAIRsharing.org is a unique registry that indexes not only databases but also provides listings of community standards and policies^62^. The policies encompass journal, funding agencies, regulatory bodies, and other organizations. The standards enumerated include terminologies, models, and schemas as well as reporting guidelines.
- **NIH data sharing repository page:** The NIH, which has had data sharing policies dating back to 2003 with the most recent one becoming effective in 2023, maintains a list of domain-specific repositories, developed by NLM through the Trans-NIH BioMedical Informatics Coordinating Committee (BMIC) effort, that are categorized into open, registered, and controlled domain repositories as well as generalist repositories^63^. This list is not meant to be exhaustive or the sole resource for selection of a repository for data sharing.
- **DOE PURE Resources:** The Office of Science from the United States (US) Department of Energy (DOE) compiles a list of data repositories, knowledge bases, analysis platforms, and other activities sponsored by the office that make data publicly available^64,65^. Specific criteria for inclusion into the list are delineated here^66^.
- **Data Repository Finder**: *Eunice Kennedy Shriver* National Institute of Child Health and Human Development (NICHD) supported the development of a repository finder tool^67^.
- **Elixir Core Resources:** Elixir, an intergovernmental organization in Europe that focuses on life science resources and infrastructure, lists a specific set of European core data resources and repositories^40^ that satisfy their criteria for a core data resource. The criteria for inclusion into the curated list are made openly available and published for the benefit of the research community.

#### Related community activities

In addition to the principles described above, there are a number of community-led initiatives that aim to generate best practices and guidance for data repositories. These community-led initiatives and conferences play an important role in promoting the use of data repositories and in developing best practices for data sharing:

- **The Research Data Alliance** **(RDA)**^4^: A community-driven global organization offering a platform to “build the social and technical bridges that enable open sharing and reuse of data.” The RDA community develops and promotes technology-neutral guidelines and provides recommendations across disciplines that transcend jurisdictional borders. The TRUST Principles^20^ is a noteworthy outcome of the RDA. In addition, the RDA has many grass-root efforts and activities aimed to implement FAIR Principles and CARE Principles. Also, the RDA has interest groups and working groups that focus on issues around repository properties, interoperability, and certifications.
- **Global Alliance for Genomics & Health (GA4GH)**^68^**:** An international community that develops and promotes policies and standards for genomic data sharing. The GA4GH community has developed the DRS (Digital Repository Service) API (Application Programming Interface), which provides machine-actionable access to data agnostic to cloud computing providers.
- **World Data Systems (WDS)**^69^**:** An international organization that develops and supports a community of scientific data repositories and related data stewardships. WDS contributes to the development of the CoreTrustSeal certification standard^36^ and endorses FAIR^18^, TRUST^20^, and CARE^21^ Principles.
- **Conferences:** In addition to community-led organizations, conferences, such as those organized by the RDA^4^, WDS^69^, and GA4GH^68^, help bring together various stakeholders to discuss issues concerning data repositories. Other conferences that address repository-relevant topics include: iPres^70^, which focuses on digital preservation; the International Society for Biocuration^22^, which hosts events on the curation of biological data and knowledge; FORCE11^71^, with events on scholarly publication communication; and the Open Repository Conference^72^, which gathers various types of repository organizations.

## Discussion

The importance of accessible, well-maintained, and efficiently operated repositories in modern biomedical research cannot be overstated. They are not only crucial for advancing biomedical research but, through robust data management practices, they significantly bolster scientific impact by facilitating data and knowledge discovery, integration, and reuse. Many repositories operate as a silo without using standard repository management practices. However, the burgeoning scale and complexity of data usage, propelled by advancements in artificial intelligence, underscores the necessity for repositories to operate in a trustworthy manner using community-based management practices and principles.

In this article we define key repository concepts and integrate community-based recommendations and principles. We also present the results of our analysis into repository metrics and lifecycle management. The paper proposes a lifecycle model for biological data repositories based on the survey of many different models, including the product lifecycle model. A unique aspect of our proposed lifecycle model is the explicit acknowledgement of an inflection point where decisions may be made based on a cost/benefit analysis resulting in a decision to reinvest in a repository or sunset it due to factors that include obsolete technology, declining use, or lack of sustainable funding. Such a model is needed to facilitate the discussion of biomedical data repository lifecycle management and sustainability.

Although we do not delve into dataset lifecycle or specific details about individual repositories and their usage, the Resources section provides links to additional information. Topics concerning the support, improvement, and sustainability of repositories are not covered in this paper, but are discussed elsewhere^73–75^. Through our analysis and survey of available metrics information we found that there are limitations in current approaches in that traditional logging analytics and bibliometrics do not fully address the need to understand the scientific and public health impacts of data repositories.

In conclusion, addressing the challenges in managing biomedical data repositories requires a unified collaborative approach among researchers, policymakers, and funding bodies. It is crucial for the community to draw upon successful standards and practices from other domains and establish a coherent framework for the future. By doing so, we can ensure that biomedical data repositories preserve their invaluable role in scientific research and continue to support data sharing, adapt to technology advancements and social needs, and contribute to global knowledge and innovation. A shared understanding of the principles, concepts, metrics, operational best practices, and lifecycle governing data repository management is fundamental to cultivating a robust biomedical repository ecosystem.

## Methods

The paper was developed through a series of foundational efforts:

1. **Working Group Discussions:** Discussions at the trans-NIH Metrics and Lifecycle Working Group meetings initiated the process, with the goal of identifying gaps in understanding data repositories essential for supporting NIH Data Management and Sharing plans. The group acknowledged a significant knowledge gap regarding data repositories and their operations, leading to the decision to produce a white paper to address these issues. To support this work, the group reviewed publications and internet documents, considered documents generated by the Research Data Alliance (RDA), engaged researchers in discussion at workshops and conferences, engaged NIH staff in discussions, and among other works, formed working definitions of repository categories.
2. **Workshops & Surveys:** Workshops were organized to engage with specific challenges and gather input from the community, promoting collaborative work. Notably, two workshops were organized that supported development of this paper: “Trustworthy Biomedical Data Repositories” and “Metrics for Data Repositories” (includes a survey for commonly used metrics from over 100 repositories; OMB Control Number: 0925–0648). Both activities produced public reports^29,76^.
3. **Repository Lifecycle Landscape Analysis and Simulations:** A landscape analysis of biomedical data repository lifecycles was carried out to support development of a NIH lifecycle model. This analysis started with the premise of the product lifecycle^77^ included an evaluation of both peer-reviewed publication and internet information and aimed to identify a) best practices for biological data repositories that are used or advocated by other organizations; b) existing definitions of data repository lifecycle; c) compare to data lifecycle models. Standard search terms, including considerations of equivalent terminology (such as repository, resource), were defined which included: Data repository lifecycle; data lifecycle decision making; data lifecycle management; discontinuing scientific data repositories; and scientific data preservation. This provided the analytical basis for the lifecycle discussion in the paper. The authors developed a biomedical lifecycle model that is similar to commercial product lifecycle models and expands on that to reflect a decision point that reflects funding and sustainability.
4. **Iterative Review and Feedback:** The draft paper was subjected to several rounds of review and feedback, incorporating perspectives from data repository experts, users, policymakers, and program administrators. This process aimed to ensure a focus on factual content over personal or organizational bias or opinion.
5. **Conferences and Feedback Solicitation:** The group used scientific conferences as a platform to present certain concepts and operations and to gather feedback, with the intention of refining and enhancing the methods presented in the paper.

## Data Availability

The current work did not produce any data.

## Code availability

The current work did not produce any code.
